# Artemisinin susceptibility in the malaria parasite *Plasmodium falciparum*: propellers, adaptor proteins and the need for cellular healing

**DOI:** 10.1093/femsre/fuaa056

**Published:** 2020-10-23

**Authors:** Colin J Sutherland, Ryan C Henrici, Katerina Artavanis-Tsakonas

**Affiliations:** Department of Infection Biology, Faculty of Infectious and Tropical Diseases, London School of Hygiene and Tropical Medicine, Keppel St, London WC1E 7HT, UK; Department of Infection Biology, Faculty of Infectious and Tropical Diseases, London School of Hygiene and Tropical Medicine, Keppel St, London WC1E 7HT, UK; Center for Global Health, Perelman School of Medicine, University of Pennsylvania, Philadelphia 19104, PA, USA; Department of Pathology, University of Cambridge, Tennis Court Rd, Cambridge CB2 1QP, UK

**Keywords:** malaria parasites, artemisinin resistance, endocytosis, haemoglobin, protein recycling, proteasome

## Abstract

Studies of the susceptibility of *Plasmodium falciparum* to the artemisinin family of antimalarial drugs provide a complex picture of partial resistance (tolerance) associated with increased parasite survival *in vitro* and *in vivo*. We present an overview of the genetic loci that, in mutant form, can independently elicit parasite tolerance. These encode Kelch propeller domain protein PfK13, ubiquitin hydrolase UBP-1, actin filament-organising protein Coronin, also carrying a propeller domain, and the trafficking adaptor subunit AP-2μ. Detailed studies of these proteins and the functional basis of artemisinin tolerance in blood-stage parasites are enabling a new synthesis of our understanding to date. To guide further experimental work, we present two major conclusions. First, we propose a dual-component model of artemisinin tolerance in *P. falciparum* comprising suppression of artemisinin activation in early ring stage by reducing endocytic haemoglobin capture from host cytosol, coupled with enhancement of cellular healing mechanisms in surviving cells. Second, these two independent requirements limit the likelihood of development of complete artemisinin resistance by *P. falciparum*, favouring deployment of existing drugs in new schedules designed to exploit these biological limits, thus extending the useful life of current combination therapies.

## Teaser

This review summarises evidence that tolerance of the antimalarial drug artemisinin by *Plasmodium falciparum* requires suppression of endocytic capture of host erythrocyte haemoglobin, an activator of the drug in early-stage parasites, coupled with an enhanced capacity for repair of cellular damage.

## OVERVIEW OF ARTEMISININ SUSCEPTIBILITY IN MALARIA PARASITES

Artemisinin-based combination therapy (ACT), co-formulating a short-acting artemisinin with a long-acting partner drug, is currently recommended for uncomplicated *falciparum* malaria. The artemisinin family of drugs is derived from naturally occurring compounds produced by the wormwood *Artemisia annua* (You-You *et al*. 1982). The widespread deployment of ACT together with other control strategies has reduced malaria morbidity and mortality, particularly in Africa (WHO 2017), but ACT efficacy against *Plasmodium falciparum* infections is threatened by reduced parasite susceptibility to both artemisinin and its partner drugs in the Greater Mekong subregion (GMS) (Noedl *et al*. 2008; Dondorp *et al*. 2009; Kyaw *et al*. 2013; Ashley *et al*. 2014; Saunders, Vanachayangkul and Lon 2014; Imwong *et al*. 2017a,b). Declining ACT efficacy against *P. falciparum* in this region raises concern that multidrug resistance may emerge in, or spread to, other malaria-endemic countries (Woodrow and White 2017), and there is some evidence of decreasing ACT effectiveness in Africa (Beshir *et al*. 2013; Henriques *et al*. 2014; Muwanguzi *et al*. 2016; Yeka *et al*. 2016; Sutherland *et al*. 2017; Kone *et al*. 2020).

Efforts to understand the mechanism of reduced artemisinin susceptibility in *P. falciparum* have focused around the *in vivo* phenotype of markedly extended parasite clearance times following a 3-day course of artesunate monotherapy, or ACT, as first described in the GMS by Noedl *et al*. (2008) and Dondorp *et al*. (2009). Quantitative estimates of parasite clearance dynamics were framed as either parasite reduction ratio (fraction of initial parasitaemia remaining after 48 h of treatment) or parasite clearance half-life (estimated from a log-linear curve of microscopically quantified parasitaemia) following artemisinin monotherapy (Ashley *et al*. 2014). Surprisingly, slow-clearing parasites *in vivo* may be adequately cleared by 7 days of artemisinin monotherapy, 3 days of monotherapy followed by 3 days of ACT or a standard 3-day course of ACT (Bethell *et al*. 2011; Kyaw *et al*. 2013; Ashley *et al*. 2014; Nyunt *et al*. 2017; Rovira-Vallbona *et al*. 2020), so this phenotype is not functionally equivalent to the resistance phenotype observed for many other drugs, including chloroquine, and is best described as significantly reduced parasite susceptibility or increased parasite tolerance.

**Figure 1.**
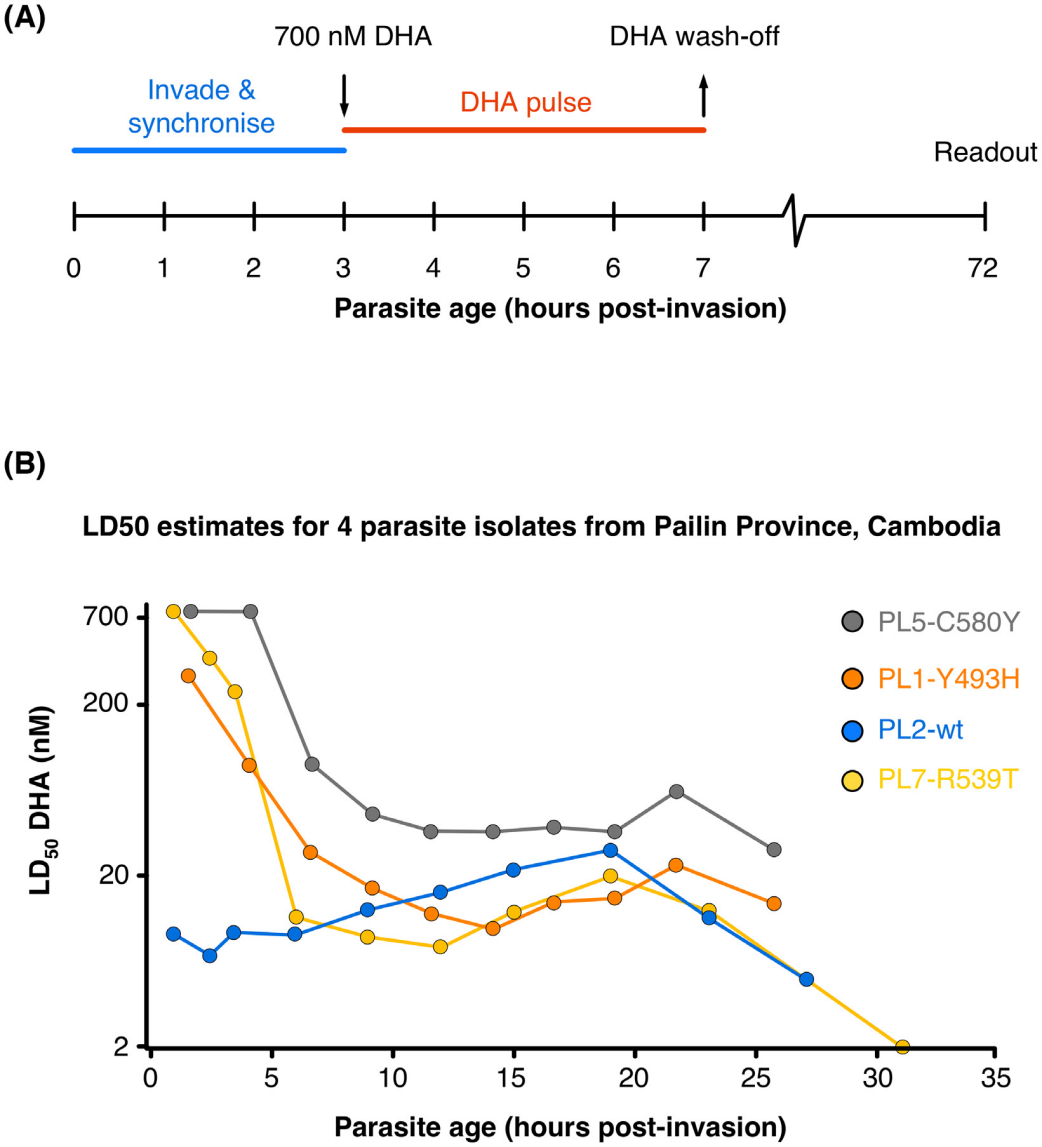
RSA for artemisinin susceptibility testing is based upon variation in drug killing across the *in vitro* cell cycle. **(A)** Schematic of timing of drug exposure and wash-off when carrying out the RSA of Witkowski *et al*. (2013). A 4-h pulse is shown, but this can vary in literature reports. **(B)** Data from Dogovski *et al*. (2015) redrawn to directly compare the DHA dose response across the first 30 h of the cell cycle *in vitro* of four parasite isolates from Pailin, Cambodia, that differ in *pfk13* genotype as indicated. This illustrates that the tolerance phenotype is only seen in the first few hours of intra-erythrocytic growth. Schizonts from all four lines were highly susceptible (LD_50_ ∼20 nM). WT—wild-type propeller domain sequence in *pfk13*.

A recurring puzzle that arose from initial research into slow-clearing *P. falciparum* in Cambodia was the poor correlation with standard *in vitro* measures of artemisinin susceptibility (Noedl *et al*. 2008; Dondorp *et al*. 2009). Witkowski *et al*. (2013) were successful in establishing a reliable, if technically challenging, *in vitro* correlate of *in vivo* clearance half-life, the ring-stage survival assay (RSA) (Fig. 1A). This method measures the fractional survival of tightly synchronised cultured early ring-stage parasites [2–4 h post-invasion (p.i.)] to a 4–6 h pulse of 700 nM dihydroartemisinin (DHA), estimated at 72 h p.i. by microscopic counting (Witkowski *et al*. 2013) or flow cytometry (Fu *et al*. 2010; Yang *et al*. 2016; Henrici, van Schalkwyk and Sutherland 2019a). Just as post-artemisinin slow clearance *in vivo* does not impart full resistance to typical treatment regimens, the reported estimates of fractional *in vitro* survival of K13 mutant parasites in the RSA vary dramatically between 2% and 45%, with laboratory protocols varying in the duration of drug exposure (Straimer *et al*. 2015; Henrici, van Schalkwyk and Sutherland 2019a, 2020). Adequate standardisation to allow robust inter-laboratory comparisons is needed and requires widely available standard parasite lines that can be shared among teams (see the section 'Detailed studies of K13 mutants *in vitro*'). Later trophozoite-stage parasites are fully susceptible to a 4-h pulse of drug exposure (Klonis, Creek and Tilley 2013; Witkowski *et al*. 2013; Dogovski *et al*. 2015; Hott *et al*. 2015; Sutherland 2017), explaining why EC_50_ estimates for DHA from standard assays with continuous 48–72 h drug exposure do not correlate with slow clearance *in vivo* (Fig. 1B). Given this observed temporally restricted partial resistance phenotype both *in vivo* and *in vitro*, we will refer to the reduced susceptibility phenotype as ‘artemisinin tolerance’ throughout this review. In our discussion, increased artemisinin tolerance is manifested as either slow clearance in the artesunate- or ACT-treated patient or higher fractional survival of parasites in the RSA *in vitro*.

The establishment by Witkowski *et al*. (2013) of an *in vitro* correlate for the slow parasite clearance phenotype directly enabled identification of the genetic basis of artemisinin tolerance in the GMS by Ariey *et al*. (2014). These authors confirmed genetic mutations encoding amino acid substitutions in the carboxy terminus of the WD40 propeller-containing protein K13 were associated with artemisinin tolerance in parasites isolated from patients. This protein, encoded by the *pfk13* locus on *P. falciparum* chromosome 13, is characterised by six tandem Kelch motif repeats that form a six-bladed β-propeller, a tertiary structure associated with protein recruitment for ubiquitin-mediated turnover (Xie, Ralph and Tilley 2020). A well-studied mammalian homologue of K13 is the human Kelch protein KEAP1, which regulates turnover of the transcription factor Nrf2 (Lo *et al*. 2006). Subsequent adaptation of Cas9 and zinc finger nuclease (ZFN) genome-editing technologies to *P. falciparum* enabled the demonstration of a subset of these mutations in the K13 propeller domain as sufficient to elicit artemisinin tolerance *in vitro* in the RSA (Ghorbal *et al*. 2014; Straimer *et al*. 2015). More recent work suggests that these mutations elicit a partial loss of function that effectively reduces the abundance of functional K13 protein in early ring-stage parasites, slowing endocytosis (see the section 'Recent functional studies of reduced ring-stage artemisinin susceptibility'). These key advances ushered in the opportunity to rigorously evaluate the associations of novel genotypes recently discovered outside the GMS, defined by distinct mutations in *pfk13* or other genes, with artemisinin-tolerance phenotypes *in vitro* (Kamau *et al*. 2015; Taylor *et al*. 2015; Menard *et al*. 2016; Muwanguzi *et al*. 2016).

Although *pfk13* mutations became a useful genetic marker for artemisinin tolerance in clinical infections, these results did not lead to any immediate unravelling of the underlying resistance mechanism. This is in part due to the complexity of artemisinin drug action. Parasite killing has been consistently linked to parasite haemoglobin metabolism, with reduction of the artemisinin endoperoxide bridge by iron in haem (Klonis *et al*. 2013), producing oxy-radical species (Heller and Roepe 2019). These radicals are thought to cause oxidative damage in the parasite cell through formation of artemisinin adducts with haem and other cellular macromolecules. Proteomic studies using artemisinin probes labelled by click chemistry reveal a large number of alkylated cellular polypeptides (Wang *et al*. 2015; Ismail *et al*. 2016), supporting the view that biomolecular adducts are formed stochastically by activated artemisinin endoperoxides (Jourdan *et al*. 2019). Two studies have also suggested that artemisinins cause rapid depolarisation of the mitochondrial membranes, potentially as a result of interaction between endoperoxide and iron–sulfur clusters (Wang *et al*. 2010; Peatey *et al*. 2015). It follows that haemoglobin uptake and breakdown is central to artemisinin activation and subsequent cytotoxicity, a proposal supported by the observation that disruption or inhibition of haemoglobin-metabolising falcipain proteases induces artemisinin tolerance *in vitro* (Klonis *et al*. 2011; Xie *et al*. 2016). However, the chemical activation of artemisinins has not been clearly visualised in live cells, and the persistence of these oxy-radical species and the limits of their diffusion through the cytosol, mitochondrial, nuclear or lysosome compartments are poorly defined.

Artemisinin activation and the mechanism of artemisinin tolerance are expected to largely involve the food vacuole (FV) of the parasite, already implicated as a site of antimalarial resistance adaptation by *P. falciparum*. In particular, mutations in the gene encoding the chloroquine resistance transporter in the FV membrane reduce susceptibility to chloroquine and piperaquine by changing drug efflux across the vacuole (Fidock *et al*. 2000; Eastman *et al*. 2011; Ross *et al*. 2018; Dhingra *et al*. 2019). In some studies, additional copies of the gene encoding another FV protease involved in haemoglobin breakdown, plasmepsin II, have been linked to reduced susceptibility to piperaquine (Silva *et al*. 1996; Amato *et al*. 2017). However, no clear cross-resistance has been demonstrated in *P. falciparum* to date between chloroquine or piperaquine and artemisinin, suggesting the mechanism of artemisinin tolerance is less about the rate of haemoglobin metabolism and haem accumulation and rather more about a requirement for haemoglobin catabolism for drug activation. Further, artemisinin tolerance is greatly increased *in vitro* by a short pulse of low temperature or heat shock, likely to briefly perturb many cellular processes, including haemoglobin uptake and protein turnover in ring stages, whereas chloroquine susceptibility is unaffected (Henrici, van Schalkwyk and Sutherland 2019a), supporting the idea that resistance mechanisms for these two drugs are independent in *P. falciparum*.

Despite its importance to intra-erythrocytic parasite growth and the action of multiple drugs, including artemisinin, the mechanism of host cell cytosol uptake and transport to the FV is poorly understood. Electron microscopy (EM) studies suggest that haemoglobin enters the asexual parasite through various endocytic processes and is first taken up into small vesicles that are absorbed into the FV (Elliot *et al*. 2008; Abu-Bakar *et al*. 2010; Milani, Schneider and Taraschi 2015). Structures involved in the uptake of host cell components into parasite cytosolic compartments include some labelled by phosphoinositide 3-phosphate (PI3P) (Jonscher *et al*. 2019). PI3P is a phospholipid component of endosomal membranes in other eukaryotes and has been implicated in K13-mediated artemisinin tolerance. K13 binds PI3P kinase (PI3K), the kinase that produces PI3P, and artificial induction of PI3P levels increases ring-stage artemisinin tolerance (Mbengue *et al*. 2015; Bhattacharjee *et al*. 2018). Recently, GFP-tagged K13 was localised to PI3P-labelled membrane compartments in close proximity to the parasite's FV (Birnbaum *et al*. 2017), and to peripheral structures bearing a strong resemblance to the parasite cytostome, a plasma membrane invagination involved in endocytosis, by 3D structured illumination microscopy (SIM) visualisation (Yang *et al*. 2019).

In eukaryotes, the process of substrate-specific endocytosis involves cargo selection and aggregation by the heterotetrameric adaptor protein 2 (AP-2) complex into clathrin-coated pits, which bud to form vesicles (Brodsky *et al*. 2001; Traub 2003; Yap and Winkler 2015; Kaksonen and Roux 2018). AP-2 is one of up to five distinct adaptor complexes that mediate endocytosis and intracellular transport (Park and Guo 2014; Yap and Winckler 2015). While AP-2 function has only recently been studied in apicomplexans (Henrici *et al*. 2020), the clathrin heavy chain and AP-1 have been localised to post-Golgi secretory structures in *P. falciparum* and the related *Toxoplasma gondii* (Ngô *et al*. 2003; Pieperhoff *et al*. 2013; Kaderi Kibria *et al*. 2015; Venogupal *et al*. 2017), which seem largely distinct from the structures labelled by PI3P and K13 in *P. falciparum*. As in other eukaryotes, AP-2 participates in endocytosis in *P. falciparum*, which surprisingly seems to be a clathrin-independent process (Birnbaum *et al*. 2020; Henrici *et al*. 2020). AP-3 and AP-4 have not yet been studied or implicated in drug responses in apicomplexans, though are likely to participate in post-Golgi transport as in other eukaryotes. Only one AP-5 subunit homologue is present in *Plasmodium* genomes studied to date, and this occurs as a pseudogene, and so AP-5 is unlikely to be functional.

Interestingly, genetic studies of AP-2 in *Plasmodium* supported a role for this complex in artemisinin susceptibility before K13 mutations were discovered. Whole-genome association studies in the rodent malaria species *P. chabaudi* first identified a mutation in the *pcap2μ* locus associated with experimentally selected artemisinin resistance (Hunt *et al*. 2007; Henriques *et al*. 2013). Our recent data indicate that *P. falciparum* expressing the orthologue of this originally discovered *P. chabaudi* mutation is tolerant to pulses of artemisinin *in vitro* (Henrici, van Schalkwyk and Sutherland 2019b), and conditional knock-sideways of AP-2µ similarly increases artemisinin tolerance (Birnbaum *et al*. 2020). Other studies have investigated the role of a distinct *pfap2μ* mutation at codon 160, identified in clinical infections in East Africa (Henriques *et al*. 2015).

These data provided the first link between endocytosis and artemisinin tolerance, and in the sections that follow we will consider in detail the proposed importance of haemoglobin uptake to artemisinin action in *Plasmodium*, the impact of interference in endocytosis on cell development and drug susceptibility, and the growing evidence that modulation of protein uptake through the cytostome–endosome network is required for parasites to survive a short exposure to DHA *in vitro*. We will then attempt to synthesise current observations into a coherent broader picture of the ways in which malaria parasites may evade artemisinin-mediated killing, including consideration of the need for surviving cells to repair any cellular damage resulting from artemisinin exposure. The implications of this understanding for future treatment strategies to prolong the useful efficacious life of current ACT chemotherapies will also be considered.

## VARIANTS OF DIVERSE PARASITE MOLECULES CAN ELICIT RING-STAGE SURVIVAL *IN VITRO*

### Detailed studies of K13 mutants *in vitro*

Initial studies of K13 polymorphisms in *P. falciparum* from the GMS with extended clearance half-lives after artemisinin treatment identified five mutations of interest (Ariey *et al*.2014; Ashley *et al*. 2014; Menard *et al*. 2016). These were in approximate order of frequency:

- C580Y (Cambodia, Vietnam, Laos, Thailand, Myanmar)
- F446I (Myanmar, China)
- R359T (Cambodia, Vietnam, Laos)
- Y493H (Cambodia, Vietnam)
- I543T (Cambodia, Vietnam)

Evidence of *in vitro* impact of these mutations on RSA survival was first provided by Ghorbal *et al*. (2014). As a proof-of-principle exemplar of the effectiveness of CRISPR-Cas9 genome editing in *P. falciparum*, these authors engineered the *pfk13* locus of wild-type laboratory line 3D7 to encode the C580Y K13 variant, derived two independent clonal lineages and demonstrated RSA survival estimates of 11.14% and 15.81%, respectively.

**Figure 2.**
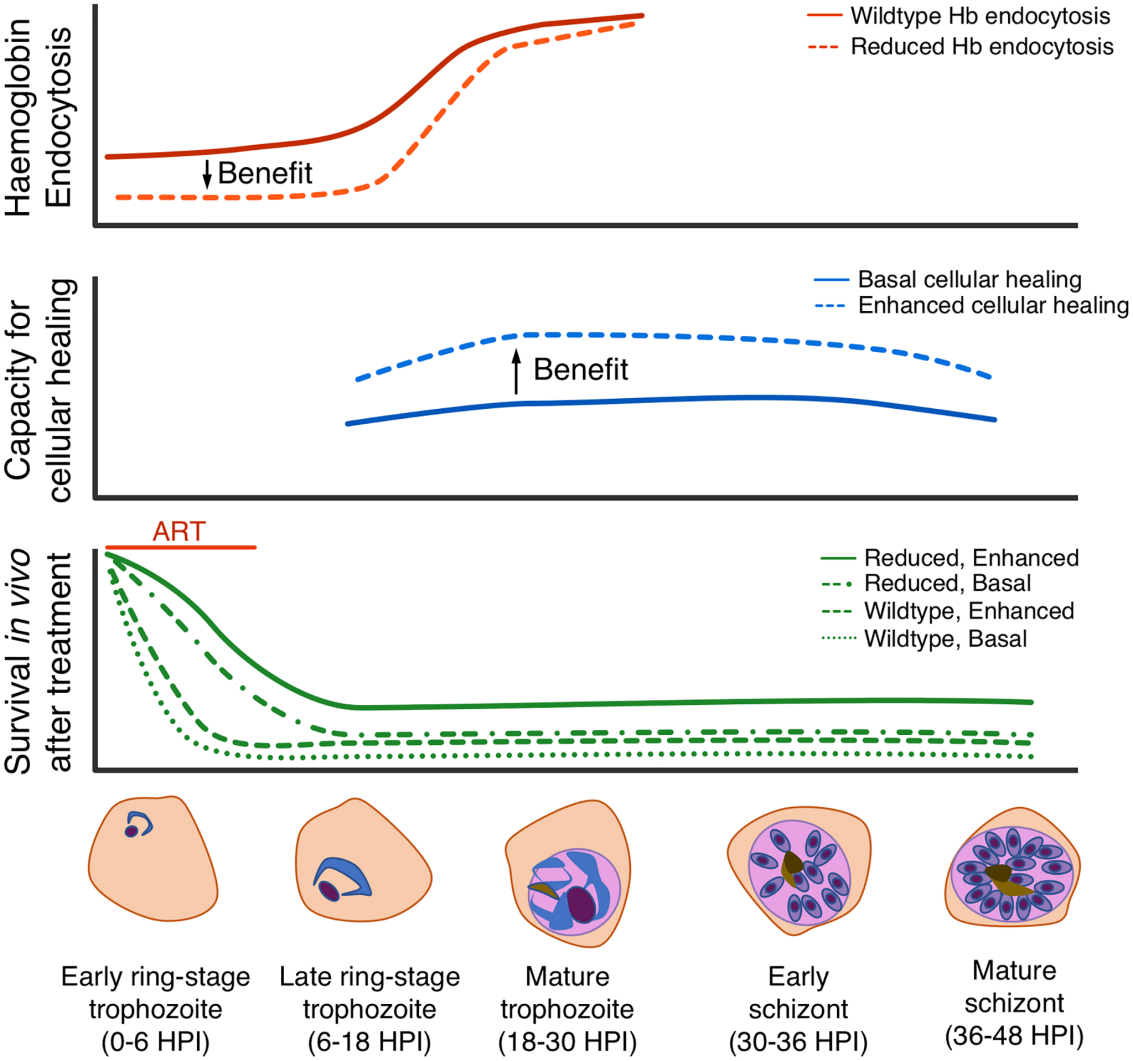
Two-component model for parasite survival of artemisinin treatment *in vivo*. Conceptual model in which two components of *P. falciparum* cellular biology (endocytosis—top panel; cellular healing capacity—middle panel) contribute to survival across 48 h of intra-erythrocytic schizogony following exposure to artemisinin treatment *in vivo* within an approximate ‘artemisinin exposure window’ during which suppression of Hb endocytosis can be advantageous (bottom panel). This simplistic representation sees both components as binary (wild-type/reduced; basal/enhanced), but this is unlikely to hold true. Only parasites encountering artemisinin during this ‘window’ (the first few hours of intra-erythrocytic development) may survive; later stages remain susceptible and are unlikely to survive whatever their phenotype (not shown). **Horizontal axis:** hours post-invasion. **Red vertical axis:** relative efficiency of Hb endocytosis up to mature trophozoite stage. The alternate state of ‘reduced Hb endocytosis’ can be generated by the presence of Kelch 13 mutations (Ariey *et al*. 2014; Straimer *et al*. 2015) that reduce the endocytic uptake (from host cytosol) of haemoglobin entering through cytostomes or other endocytic structures (Yang *et al*.2019). The arrow shows direction of benefit, which is only exerted in ring stages. The same alternate state can be reached by expression of variants of PfCoronin (Demas *et al*. 2018), AP-2m, UBP-1 (Henrici *et al*. 2020), disruption of falcipain-2a (Klonis *et al*. 2011; Xie *et al*. 2016) and hypo- or hyper-thermal pulses at early ring stage (Henrici *et al*. 2019a). **Blue vertical axis:** capacity for cellular healing through adaptive modification of the proteasome, the unfolded protein response (UPR), chaperone-mediated protein re-folding, autophagy and DNA repair; the arrow shows direction of benefit. These phenotypes have not been adequately characterised to allow a precise prediction of impact, and any particular parasite may have enhanced capacity in only some, but not all, of these functions. It is assumed these functions are most active prior to schizogony and segregation of merozoites. Cellular healing capacity is encoded independently of ring-stage tolerance, is thought to be multigenic and may also require epigenetic adaptations. Therefore, it is likely to be rarely achieved, and may come at a cost to overall reproductive fitness in the absence of intense drug pressure. **Green vertical axis:** relative survival capability in a treated host of parasites with each of the four combined states possible in a simplistic binary model for each of the two components—haemoglobin endocytosis and capacity for cellular healing, respectively. In contrast to *in vitro* assays, damaged parasites surviving the artemisinin exposure window may be unlikely to survive host clearance mechanisms without an enhanced cellular healing phenotype. Only the effects of an artemisinin encounter at early ring stage are shown.

Straimer *et al*. (2015) then presented a major analysis of four of the K13 variants from the GMS—C580Y, R539T, Y493H and I543T—as well as the M476I variant originally described in an experimentally derived artemisinin-tolerant line of Tanzanian origin by Ariey *et al*. (2014), which also occurs at low frequency in Myanmar (Nyunt *et al*. 2015). In a classical application of reverse genetics, deploying ZNF genome editing, Straimer and colleagues demonstrated three important features of K13-mediated artemisinin tolerance *in vitro*. First, by reverting the endogenous K13-encoding gene back to wild type in four different artemisinin-tolerant lines, the main GMS variants were shown to be sufficient to induce artemisinin tolerance *in vitro* by the RSA (Straimer *et al*.Fig.   2A–D). Second, when introduced into the same genetic background (chloroquine-resistant laboratory line Dd2), each of the five K13 variants elicited significantly differing levels of survival in the RSA (Fig. 2J; ibid.). Third, when introduced into five different parasite lines, the C580Y variant elicits five different fractional survival estimates in the RSA (Fig. 2K; ibid.), clearly demonstrating that genetic background has a profound effect on the intensity of the tolerance phenotype *in vitro*. Additionally, an important contribution of this work was the establishment and *in vitro* phenotypic characterisation of three isogenic parasite lines of Cambodian origin, namely the slow-clearing *in vitro* tolerant patient isolate Cam 3.II, which harbours the R539T variant of K13, and Cam 3.II^REV^ (genome-edited to R539R) and Cam 3.II^C580Y^ (genome-edited from Cam 3.II^REV^). This well-characterised isogenic trio, made widely available, should serve as international standard for inter-laboratory calibration and comparison of RSA outputs (Henrici, van Schalkwyk and Sutherland 2019b).

### PI3P and PI3K in K13-mediated artemisinin tolerance

Mbengue *et al*. (2015) provided some of the first mechanistic data underlying the phenotype of artemisinin tolerance associated with K13 mutant genotypes. Specifically, these authors reported that the small phospholipid PI3P and the associated PI3K were direct targets of artemisinin. It was also observed that expression levels of PI3K and PI3P differed in parasites expressing variant K13. Unlike higher order eukaryotes, the *P. falciparum* genome apparently encodes only one PI3K enzyme (PfPI3K), and in lineages expressing K13^C580Y^ or K13^R359T^, the authors found significantly higher levels of PI3K, potentially linking this factor to RSA survival. In mammalian cells, the closest K13 orthologue is a ubiquitylation scaffolding factor, and the authors propose that K13 regulates PI3K levels and thus the abundance of PI3P. However, it remains unclear how this high-level expression might functionally contribute to the artemisinin tolerance phenotype, or whether it is a cellular adaptation to reduced endocytic activity.

In many other eukaryotes, PI3P is a component of the early endolysosomal system—the initial destination of endocytic trafficking (Gillooly *et al*. 2000; Gillooly, Simonsen and Stenmark 2001). Mbengue *et al*. (2015) reported PI3P at the endoplasmic reticulum (ER) in young ring-stage parasites, and that treatment with DHA relocates PI3P to the parasitophorous vacuole, a result recapitulated by subsequent work (Bhattacharjee *et al*. 2018). This localisation, and thus the mechanistic interpretation of PI3P in artemisinin tolerance, is seemingly at odds with several other studies (McIntosh *et al*. 2007; Tawk *et al*. 2010; Boddey *et al*. 2016; Ebrahimzadeh, Mukherjee and Richard 2018; Jonscher *et al*. 2019; Birnbaum *et al*. 2020), likely in part because of differences in reporter protein used to localise PI3P in the cell. Mbengue *et al*. detected PI3P via overexpression of the FYVE domain of EEA1, a Rab5-dependent PI3P-binding protein found in other eukaryotes, that mostly labels the early endosome where Rab5 is abundant. In *Plasmodium*, the localisations of Rab5 family members are not well articulated, and thus the labelling of PI3P with EEA1 is of uncertain meaning. Other studies in eukaryotes deploy the mammalian protein p40phox, a specific PI3P-binding factor, to avoid dependence on the EEA1 FYVE domain for PI3P localisation. In *Plasmodium*, P40phox sensing of PI3P reproducibly demarcates the FV and apicoplast membranes and labels membranes of vesicles containing host cell (haemoglobin-containing) cytosol (Ebrahimzadeh, Mukherjee and Richard 2018), rather than the ER as suggested by Mbengue and Bhattacharjee. In particular, Birnbaum *et al*. (2020) demonstrate using P40phox sensing that K13 is very closely apposed to the PI3P-labelled FV and vesicular membranes and so association with these structures cannot be ruled out. A recent ultrastructural study reported K13 at the cytostome, the structure from which host cell cytosol-containing vesicles likely bud (Yang *et al*. 2019). However, this group did not examine the localisation of K13 with respect to PI3P reporters. Interestingly, while the role of PI3P in the story of K13-mediated artemisinin tolerance remains unclear, several proteomic datasets comparing isogenic wild-type and mutant K13 lineages have been published over the last several years, and none have identified changes in PfPI3K abundance or a direct interaction between K13 and PfPI3K (Siddiqui *et al*. 2017; Birnbaum *et al*. 2020; Gnädig *et al*. 2020). Using docking studies and molecular dynamics simulations, Mbengue *et al*. (2015) suggested that ART directly inhibits PfPI3K via non-covalent H-bonding with D1889 and Y1915 side chains, but no further data have emerged supporting this view, and Hassett *et al*. (2017) report clear evidence that functional inhibition of purified PI3K requires covalent binding of activated endoperoxide and occurs randomly across many molecular sites. Experimental results discussed above (see the section 'Overview of artemisinin susceptibility in malaria parasites') show that activated DHA primarily acts through formation of covalent biomolecular adducts by indiscriminate oxidation of macromolecules in close proximity. As a major component of the FV and endosomal/vesicular membranes, increased PI3P abundance in K13 mutant parasites may be related to a lag in formation of these bi-layered structures, which are clearly required for asexual survival. Further functional studies are required to verify a direct role in artemisinin tolerance of this lipid and its kinase partner.

### Endocytic adapter protein subunit AP-2μ

The mechanisms by which the *P. falciparum* parasite internalises and transforms haemoglobin into haemozoin are central to the efficacy of many antimalarial drugs, and haem-derived iron is known to catalyse the oxidative opening and activation of the artemisinin endoperoxide moiety. Although direct inactivation of falcipain 2a in the laboratory increases artemisinin tolerance (Klonis *et al*. 2011; Xie *et al*. 2016), mutations in haemoglobinases or FV transporters have not been identified as causative for artemisinin tolerance in human infections or laboratory-evolved lineages (Ariey *et al*. 2014). Hunt *et al*. (2007) derived artemisinin-tolerant lineages in the rodent malaria parasite *P. chabaudi*, and subsequent whole-genome sequencing revealed a mutation in the medium subunit of the canonical eukaryotic endocytic trafficking complex AP-2, invoking the possibility that haemoglobin uptake rather than digestion might manipulate artemisinin tolerance (Henriques *et al*. 2013).

Subsequent studies in human *P. falciparum* infections found evidence of directional selection on *pfap2μ *genetic variants during artemisinin treatment (Henriques *et al*. 2014), and recent reverse genetics experiments confirmed that AP-2µ does mediate artemisinin tolerance. Specifically, Henrici, van Schalkwyk and Sutherland (2019b) demonstrated that when installed into *P. falciparum*, the I592T mutation, orthologous to that first described in artemisinin-resistant *P. chabaudi* (*pcap2μ *I568T), increases RSA survival. Birnbaum *et al*. (2020) further demonstrated in *P. falciparum* that AP-2µ inactivation via conditional knock-sideways also causes ring-stage tolerance to artemisinin, suggesting that the *P. chabaudi* mutation causes an impairment of AP-2µ function. This is consistent with lower concentrations of haemoglobin-derived peptides found in artemisinin-tolerant parasites compared with artemisinin-sensitive parasites (Siddiqui *et al*. 2017), supporting a role for endocytosis in artemisinin tolerance and AP-2 therein. Further evidence that the AP-2 adaptin complex mediates ring-stage artemisinin susceptibility in *P. falciparum* is provided by the laboratory evolution of a parasite lineage with a measurable tolerance phenotype found to harbour a non-synonymous change in the AP-2α subunit, though a reverse genetics validation of this mutation is outstanding (Rocamora *et al*. 2018).

### UBP-1

UBP-1 was first implicated in drug tolerance in *P. chabaudi* (Hunt *et al*. 2007). Growth under sequential and sustained chloroquine, artemisinin and artesunate pressure *in vivo* resulted in the appearance of non-synonymous point mutations within the coding sequence of the putative UBP-1 locus in two clones, as well as mutations in the locus encoding AP-2µ. Experimental introduction of the orthologous *pcubp1* mutations into *P. falciparum* (encoding V3275F, V3306F; Henrici *et al*. 2019) followed by DHA treatment confirmed an *in vitro* ring-stage phenotype in both species for the V3275F mutant, but not the V3360F mutant, experimentally validating the involvement of UBP-1 in artemisinin tolerance in *P. falciparum*. Transgenic *P. berghei* parasites expressing mutations orthologous to V3275F and V3306F recrudesced earlier than wild-type parasites after artesunate challenge (Simwela *et al*. 2020), confirming UBP-1 mediates ART tolerance *in vivo*, although the *in vivo* susceptibility assays deployed by Simwela *et al*. are not directly comparable to outputs of the RSA for *P. falciparum in vitro*. The orthologue of PfUBP-1(V3275F) was found to recrudesce faster than the orthologue of PfUBP-1(V3306F). Interestingly, the latter mutation also increased chloroquine tolerance in *P. berghei*, but this is not the case in *P. falciparum* (Henrici, van Schalkwyk and Sutherland 2019b).

Amino acid substitutions within this gene have also been identified in naturally occurring infections. In cultured *P. falciparum* isolates from Kenyan children, *pfubp1* mutations outside of the ubiquitin C-terminal hydrolase (UCH) domain were associated with higher DHA EC_50_ estimates by genome-wide association analysis, and these isolates displayed decreased genetic variation in a region of chromosome 1 centred on *pfubp1* (Borrmann *et al*. 2013), consistent with directional selection in the field. In a separate study of Kenyan children, these same mutations were statistically associated with ACT treatment failure, though they have not been studied *in vitro* (Henriques *et al*. 2014). Moreover, genomic surveillance of GMS field isolates linked an additional point mutation within the UCH domain, encoding R3138H, with ART resistance (Cerquiera *et al*. 2017), which was recently validated in the RSA (Birnbaum *et al*. 2020).

Despite the genetic evidence for involvement of UBP-1 in mediating ART tolerance, the protein remains poorly characterised. The *pfubp1* locus encodes a large, 415 kDa protein with the putative UCH domain at its carboxy terminus. Although UBP-1 has been classified as a deubiquitinating (DUB) enzyme on the basis of this domain, its functionality in mediating ubiquitin hydrolysis is hypothetical, and the potential function of the larger upstream portion of this protein has not been explored. PiggyBac mutagenesis studies and conditional knock-sideways experiments have identified UBP-1 as essential and involved in haemoglobin endocytosis (Zhang *et al*. 2018; Birnbaum *et al*. 2020); however, these data do not reveal what segment of this protein is necessary for parasite viability nor do they provide any clues as to its specific function. Interestingly, knock-sideways of UBP-1 in the ring stage also induces ART tolerance (Birnbaum *et al*. 2020). Thus, the mutations validated so far *in vitro* (V3275F and R3138H), which occur in the UCH-type DUB domain, seem likely to functionally disrupt the protein and its putative DUB function. UBP-1 has not been explored in other apicomplexans but USP7, a homologue of UBP-1, is involved in regulating endocytosis and surface glycoprotein abundance in the distant trypanosomatid protist *T. brucei* (Zoltner *et al*. 2015).

### Actin-associated propeller domain protein Coronin

K13 and AP-2μ were identified as proteins of interest using experimental artemisinin selection of well-established laboratory parasite lines (see above). Demas *et al*. (2018) examined whether artemisinin tolerance could arise *de novo* in African parasite populations and so subjected two recently derived parasite isolates from Senegal to long-term *in vitro* selection with increasing concentrations of DHA. Two parasite clones were isolated, one from each original isolate, that exhibited enhanced survival in the ring-stage survival assay. Among 10 mutations in seven different genes in these two clones, the gene encoding PfCoronin, a WD40 propeller domain-containing actin-binding protein, accrued mutations in both independent selections. For validation, Cas9 editing of *pfcoronin* to introduce the mutations of interest into the susceptible parental parasites increased fractional survival in the RSA in both cases. As seen for K13 and AP-2μ, EC_50_ estimates for the transgenic parasites indicated full susceptibility under continuous 72 h drug exposure in a standard assay, in contrast to the DHA-tolerant phenotype observed in the RSA (Demas *et al*. 2018). Thus, like K13 mutants, variants of PfCoronin only impart artemisinin tolerance in the early ring stage of the intra-erythrocytic cycle.

The discovery of this additional protein able to modulate artemisinin tolerance should provide new insights into the molecular mechanisms of resistance. The WD40 repeat domain of PfCoronin encodes seven blades and the protein is implicated in filamentous actin organisation in *Plasmodium* (Olshina *et al*. 2015) and other Apicomplexa. Coronin is specifically thought to organise actin filaments required for endocytosis and recycling of membranes in *Toxoplasma* (Salamun *et al*. 2014). Thus, vesicular trafficking may be a common functional platform that links K13, AP-2μ and Coronin to mechanisms of artemisinin tolerance in ring-stage parasites (Henrici and Sutherland 2018). As the experimental selection studies that identified Coronin were carried out in recently isolated African parasite isolates, it may be that *pfcoronin* mutants could emerge as components of a K13-independent artemisinin tolerance mechanism in natural parasite populations.

## RECENT FUNCTIONAL STUDIES OF REDUCED RING-STAGE ARTEMISININ SUSCEPTIBILITY

Several studies independently investigating the biological mechanisms of artemisinin tolerance were published in 2019 and 2020, providing a complex picture of the cellular adaptations that can elicit artemisinin tolerance in ring-stage *P. falciparum* parasites *in vitro*.

### K13 associates with the parasite cytostome

Yang *et al*. (2019) measured protein turnover in K13 mutant and wild-type parasites *in vitro*, demonstrating that tolerance was the result of partial loss of K13 function and providing new evidence of localisation to peripheral structures in the cell that correspond to the parasite cytostome. Experimental mislocalisation of wild-type K13 to the nucleus led to 20% survival in their modification of the RSA, and reduced abundance of haemoglobin-specific peptides (products of catabolism) in ring-stage parasites, suggestive of reduced haemoglobin uptake. This is consistent with the proposed involvement of cytostomes, specialised but poorly understood parasite structures that mediate early events in haemoglobin uptake. Specifically, each cytostome is a deep invagination of the parasite plasma and parasitophorous vacuole membranes. Red cell cytosol is expected to fill this space, and endocytic vesicles containing host cytosol are believed to bud from the cytostome and deliver haemoglobin to the digestive vacuole (DV) for degradation into amino acids. Using super-resolution and EM, Yang *et al*. identified K13 in ring-shaped peripheral structures with a cross-sectional diameter that corresponds to the average diameter of the neck of cytostomes, presumably forming a type of collar that could modulate haemoglobin uptake. This electron-dense cytostomal collar has been previously described as anchored to organising actin filaments implicated in the trafficking of haemoglobin to the FV (Lazarus, Schneider and Taraschi 2008; Smythe, Joiner and Hoppe 2008). Interestingly, as discussed above, variants of the actin-binding protein PfCoronin also induce artemisinin tolerance *in vitro* (Demas *et al*. 2018).

In addition, Yang *et al*. (2019) examined protein turnover, cellular structures and responses after short pulses of artemisinin exposure. After DHA was applied and wild-type K13 mislocalised to the nucleus, there was less overall protein damage, presumably because of impaired endoperoxide activation, due to the requirement for haemoglobin uptake to activate DHA (Klonis *et al*. 2011). In contrast to a previous work (Zhang *et al*. 2017), Yang found that wild-type and mutant K13 lineages initially respond similarly to ring-stage ART-induced stress at the level of protein translation, suggesting no intrinsic differences in translation-dependent oxidative stress response. However, the K13 mutant parasite line Cam3.II^R359T^ resumed protein turnover several hours after DHA exposure, whereas wild-type Cam3.II^REV^ did not despite similar basal levels of protein turnover in the two treated groups. Yang *et al*. argue this is evidence against K13-mediated latency being a key factor in artemisinin tolerance in favour of an intrinsic difference in artemisinin activation, although we add the obvious caveat that very few Cam3.II^REV^ parasites survived the DHA pulse, making this comparison difficult to interpret. We speculate that the observation of an extended duration of the parasite ring stage, observed in propeller domain mutant parasites (Hott *et al*. 2015), may be a direct result of a lower abundance of K13 reducing haemoglobin uptake and catabolism and so starving the ring-stage parasite of key nutrients, rather than the result of an acquired dormancy strategy.

### K13 defines a clathrin-independent endocytic compartment

In unrelated work, Birnbaum *et al*. (2020) probe the localisation and function of K13 and provide the most in-depth mechanistic interrogation of artemisinin tolerance to date, ultimately supporting the centrality of impaired endocytosis. By imaging an endogenously tagged transgenic K13-GFP (green fluorescent protein) lineage, the authors show that K13 is localised to a cytosolic compartment near the DV and apicoplast that does not colocalise with conventional, studied *Plasmodium* organelles but is also labelled by the AP-2 complex, in particular the AP-2µ subunit. Using a fused K13-biotin ligase construct, the authors further define the components of this compartment to include several previously uncharacterised factors (now labelled KIC1–KIC10), the canonical endocytic factor Eps15 and, interestingly, UBP-1. Conditional ring-stage inactivation of these factors reduces parasite uptake of fluorescent dextrans hypotonically loaded into the RBC cytosol and induces ring-stage artemisinin tolerance. These findings suggest that this compartment is intimately associated with endocytosis, a process that drives artemisinin activation in the first few hours post-invasion. Curiously, this compartment was shown to be devoid of clathrin, the canonical AP-2-interacting endocytic coat protein (see the section 'AP-2μ associates with a clathrin-independent compartment and is essential for schizogony'). Similarly, expression of the K13^C580Y^ mutation, widely circulating in the GMS and elsewhere, also reduced endocytic uptake. Reduced endocytic dextran uptake in the K13^C580Y^ mutant was not attributable to mislocalisation or altered interactions but rather to an overall reduction in K13 protein abundance, in agreement with studies linking artemisinin-resistant parasites with lower levels of K13 protein (Siddiqui *et al*. 2017; Yang *et al*. 2019). Exogenous overexpression of either the wild-type or mutant allele ablates ring-stage ART tolerance in a lineage endogenously expressing K13^C580Y^, further suggesting the phenotype elicited by naturally occurring K13 mutations is related to the amount of functional protein present.

Whereas K13 itself appears to only be essential during ring stage, inactivation of its interactors Eps15, UBP-1 and KIC7 is detrimental to parasite growth across all stages of development. The disruption of UBP-1, in particular, was shown to significantly reduce haemoglobin uptake and increased drug tolerance as determined by RSA (Birnbaum *et al*. 2020). Introduction of UBP-1^R3138H^, a naturally occurring mutation linked to ART resistance in field samples, resulted in the same effects. These results would suggest that this arginine-to-histidine change inhibits UBP-1 function, which is consistent with the observation that it was not possible to introduce two simultaneous mutations in the UCH domain of *pbubp1* (Simwela *et al*. 2020). It will be interesting to determine whether the R3138H mutation, and possibly the V3275F mutation identified in *P. chabaudi* by Hunt *et al*. (2007), results in protein instability and lower levels of UBP-1 in the cell (as seen with K13 propeller mutants). Alternatively, as they fall within or in close proximity to the UCH domain, these point mutations may directly disable ubiquitin hydrolase activity but leave overall protein abundance unaltered.

With advanced EM imaging, Birnbaum *et al*. (2020) demonstrated that the compartment labelled by K13, AP-2µ, Eps15, UBP-1 and the other KICs is likely to be the cytostome, in agreement with the data presented by Yang *et al*. (2019). Overall, the authors suggest that, unlike for canonical DV-active antimalarials like chloroquine, the mechanism of tolerance to artemisinins in field isolates is upstream of the DV and likely related to the rate of haemoglobin transport to the DV, and the availability of substrate haem-derived iron for ART activation, rather than the rate of haemoglobin digestion and haem accumulation. Interestingly, this also suggests that while potentially many mutations in diverse genes could mediate ring-stage artemisinin tolerance, development of complete ART resistance is unlikely to be mediated by variant components of this endocytic compartment alone as haemoglobin is fundamentally required for parasite growth.

### AP-2μ associates with a clathrin-independent compartment and is essential for schizogony

In a third unrelated work, Henrici *et al*. (2020) probe the localisation and function of AP-2µ, one of the core factors implicated in the genetics of artemisinin tolerance, as a window to understanding endocytosis in the wild-type parasite in the absence of artemisinin pressure. AP-2µ was expected to be localised with clathrin to peripheral membranes, but instead these factors localised to distinct surfaces, in agreement with the results of Birnbaum *et al*. (2020). Clathrin was instead found at punctae distributed throughout the cytosol, whereas AP-2µ labelled fewer discrete structures at the plasma membrane and adjacent to the FV. These AP-2µ-labelled structures were present throughout the entire asexual life cycle and in the mature schizont segregated into daughter merozoites to be carried into the nascent parasites of the next generation, strikingly colocalising with K13 across each developmental stage in good correspondence with the findings described above. EM identified AP-2µ at the plasma membrane as well as cytosolic vesicles labelled with the GTPase cofactor Rab5B, which has been previously implicated in intracellular transport at the plasma membrane and food-vacuole membrane in *P. falciparum* (Ezougou *et al*. 2014).

Conditional DiCre-mediated genetic deletion of AP-2µ was lethal and broadly disrupted schizont maturation, causing accumulation of lipids and free haemozoin in the parasite cytosol and tearing of parasite membranes, consistent with underlying fragmentation of the FV and disruption of haemoglobin uptake and lipid mobilisation or recycling. Interestingly, no deposits of host cell cytosol were visualised in any imaging studies, as had been seen in VPS45 knock-sideways experiments (Jonscher *et al*. 2019), probably because disruption of transport by VPS45 mislocalisation occurs after vesicles have formed and been internalised, in contrast to the complete blockade of vesicle formation by AP-2µ knockout.

Co-immunoprecipitation experiments performed by Henrici *et al*. (2020) identified several key factors supporting a role for the AP-2 complex in a clathrin-independent endocytosis, namely several small trafficking-associated regulators and plasma membrane factors copurified with AP-2µ including PfEHD, an epsin homology domain-containing factor previously associated with endocytic machinery in *Plasmodium* (Thakur *et al*. 2015). PfEHD contains an N-terminal dynamin-like domain, which typically drives membrane scission during vesicular budding. Though dynamins are poorly characterised in *Plasmodium*, Dynasore, a small-molecule inhibitor of dynamin GTPase activity, has been demonstrated to interfere with haemoglobin uptake in *Plasmodium* (Zhou *et al*. 2009).

Consistent with the K13 interaction studies performed by Birnbaum *et al*. (2020), K13 did not copurify as an interactor of AP-2µ despite their colocalisation. However, KIC7 (Kelch13-interacting protein 7; Birnbaum *et al*. 2020) was enriched as an AP-2µ interactor in these co-immunoprecipitations (Henrici *et al*. 2020). Thus, KIC7 may be a bridging adaptor between K13 and the AP-2 complex at sites of endocytosis. Unexpectedly, another Kelch domain-containing factor encoded on chromosome 10 (K10) also copurified with AP-2µ, and its interaction was validated by reciprocal pulldown (Henrici 2018). This network corroborates a role for endocytosis in artemisinin tolerance as manipulations of KIC7 and AP-2µ directly cause artemisinin tolerance *in vitro*, and K10 has been epistatically associated with K13-mediated artemisinin resistance in Asian parasite isolates (Cerqueira *et al*. 2017). Lastly, Henrici *et al*. (2020) defined the remaining AP-2 complex subunits in *P. falciparum* including AP-2α, which has been suggested to mediate artemisinin tolerance in a laboratory-evolved lineage (Rocamora *et al*. 2018). Importantly, neither clathrin heavy chain nor clathrin light chain was identified in the AP-2µ pulldowns, and AP-2 subunits were not identified in a reciprocal clathrin heavy chain immunopurification, further defining artemisinin tolerance-associated haemoglobin endocytosis as a clathrin-independent process (see the section 'K13 defines a clathrin-independent endocytic compartment'; Binrbaum *et al*. 2020).

### Artemisinin tolerance is linked to functional protein dose in K13 mutant lineages

Gnädig *et al*. (2020) took a different approach to elucidating K13 cellular function and the basis of artemisinin tolerance. Unlike the three studies described above, in which *cis-*tagging of endogenous K13 and AP-2µ with haemagglutinin or GFP reporters was deployed in immunofluorescence analyses of protein localisation and in co-immunoprecipitation approaches to identify interacting or proximal proteins, a panel of anti-K13 monoclonal antibodies (mAb) was developed and deployed in parallel with reporter tagging. K13 cellular distribution was examined in isogenic Cam3.II^REV^ and Cam3.II^R539T^ lines and a pair of lines from the artemisinin-susceptible NF54 background edited to express either wild-type K13 or the C580Y tolerance-associated variant. Interestingly, overexpressing wild-type K13 in either mutant background generated a pseudodiploid genotype in which the artemisinin tolerance phenotype was greatly reduced or completely ablated. This indicates that K13-mediated artemisinin tolerance is not a dominant phenotype and that the R539T and C580Y mutations do not behave as dominant Mendelian alleles. Instead, these findings support the conclusion of Yang *et al*. (2019) that K13-mediated artemisinin tolerance results from a lower dose of functional K13 in the cell as mutations cause a partial loss of function. Gnädig *et al*. (2020) estimate this to be functionally equivalent to a 50% reduction in protein abundance in parasites carrying the K13^R359T^ allele compared with wild type, similar to the ∼30–50% reduction reported by Birnbaum *et al*. for K13^C580Y^-expressing parasites, corresponding to an ∼30–40% reduction in functional endocytosis (Birnbaum *et al*. 2020; Gnädig *et al*. 2020). Consistent with these observations, Heller, Goggins and Roepe (2018) demonstrated that ferriprotoporphyrin IX-artemisinin adducts were significantly less abundant in artemisinin-tolerant parasites with K13 mutations than in isogenic wild-type parasites.

Immunofluorescence and immunoelectron microscopy (IEM) experiments by Gnädig *et al*. (2020), using their anti-K13 mAb as well as parasite lines with tagged *pfk13* alleles, reported a localisation consistent with the colocalised distributions of AP-2µ-3xHA and K13-GFP described by Henrici *et al*. (2020). K13 was reported in a single cytosolic focus during the first few hours post-invasion, and this was observed to replicate and segregate to daughter merozoites during schizogony. Super-resolution imaging confirmed that K13-labelled structures resemble donuts or tubes, which could represent the cytostome location described by Yang *et al*. (2019) and Birnbaum *et al*. (2020). IEM using these monoclonal reagents frequently identified K13 in small membrane-bound structures near the ER, FV and plasma membrane, sometimes associating with structures likely to represent cytostomes. No differences in localisation were observed between K13 wild-type and mutant parasites in the absence of drug pressure. However, when exposed to a 6-h ring-stage challenge of 700 nM DHA, wild-type K13 remained partly colocalised with several endocytosis-associated Rab5-family GTPases, including Rab5B as reported for AP-2µ by Henrici *et al*. (2020), Rab6 and Rab7, proteins expected to participate in post-Golgi transport near the DV (Siddiqui *et al*. 2020). The mutant K13 molecule, on the other hand, became less closely associated with Rab interactors, potentially as a consequence of its reduced stability and DHA-induced cytosolic oxidation.

To identify factors interacting with K13, Gnädig *et al*. (2020) performed co-immunoprecipitations using their anti-K13 MAb, and generated a complex dataset of potential functional partners, using both wild-type and K13^R539T^ parasite lines. Interactors included a significant selection of Rab family GTPase proteins, recognised as regulators of targeting and fusion of trafficking vesicles involved in secretion and endocytosis (Langsley *et al*. 2008) and partly colocalised in the imaging studies. Consistent with the findings of Henrici *et al*. (2020), the list of interacting factors did not include AP-2μ and included only a single protein (PFK9, gene ID: PF3D7_0915400) that also occurs in the list of K13 interactors produced by Birnbaum *et al*. (2020). While Birnbaum *et al*. (2020) fused K13 to a minimal biotin transferase to label nearby and interacting proteins, it is striking that such an apparently promiscuous methodology did not isolate more factors in common with the interactors identified by Gnädig *et al*. (2020). An important difference between these methodologies is that whereas the bio-ID approach operates in live cells before lysis, immunopurification relies on the preservation of native interactions during cell lysis and adequate solubility in the chosen lysis buffer. Gnädig *et al*. (2020) did not identify UBP-1 or any KIC-family proteins as interacting partners of K13 nor, in agreement with previous studies, did they copurify PI3K. Overall, the data presented are consistent with an important role of K13 in modulating haemoglobin uptake and vesicular transport via the cytostome and Rab network. Surprisingly, these experiments also suggest an unexpected involvement of the mitochondria, particularly in the mutant K13 response to artemisinin pressure, as a number of mitochondrial proteins were identified as putative interacting partners. Gnädig *et al*. propose that K13 is specifically recruited to the mitochondrion. However, permeabilisation of the mitochondrion and loss of membrane polarisation following artemisinin exposure have been previously described (Wang *et al*. 2010; Peatey *et al*. 2015), and this also occurs in all dying cells, and so may lead to inappropriate ingress of proteins from other compartments. Given the only partial concordance of these findings with those of Birnbaum *et al*. (2020), further studies of K13 and mitochondrial function in *P. falciparum* are of importance to clarify the impact of treatment and acquired tolerance on the parasite organelle.

## OXIDATION AND PROTEOTOXICITY—THREATS TO *IN VIVO* FITNESS OF ARTEMISININ-TOLERANT PARASITES

### Artemisinin-tolerant parasites have activated stress responses and repair mechanisms

Prior to the identification of *pfk13* as the major genetic determinant of slow parasite clearance in artemisinin-treated malaria patients in the GMS, detailed genomic and transcriptomic studies had provided evidence that parasites displaying this phenotype were characterised by an enhanced cellular stress response. Slow-clearing patient isolates harboured specific variants of genes involved in DNA synthesis, mismatch repair and oxidative damage response (Miotto *et al*. 2013; Takala-Harrison *et al*. 2013), and showed upregulation of stress response factors in the ring stage and of ring-stage transcripts in otherwise trophozoite-stage cells, despite an overall reduction in global transcription (Mok *et al*. 2011). Transcripts that were significantly upregulated in slow-clearing patient isolates included cyclophilin19B, dolichyl-phosphate-mannose protein mannosyltransferase, ER-resident calcium-binding protein, BiP/grp78, and a protein disulfide isomerase (Mok *et al*. 2015), but this group of molecules, associated with the unfolded protein response (UPR), was highly transcribed generally in parasites from the GMS compared with other countries of origin in this dataset. More recently, lineages expressing K13 mutations were found to have abnormally elevated early ring-stage levels of the phosphorylated form of eIF2α, a translationally repressive stress response factor that may play a role in recovery from artemisinin-induced damage (Zhang *et al*. 2017; Yang *et al*. 2019). Puzzlingly, this phosphorylation event seems to be independent from K13 activity, as conditional inactivation of K13 in early ring stages has no effect on eIF2α-P levels (Yang *et al*. 2019). Other proteomic and transcriptomic studies have revealed increased expression of stress response factors in K13 mutant ring-stage parasites, suggesting a proteostatic mechanism may account for a delay in early cell development (Mok *et al*. 2015; Siddiqui *et al*. 2017; Gnädig *et al*. 2020) and that subsequent survival post-artemisinin exposure requires enhanced protein turnover and oxidative damage response.

Further evidence supporting this view comes from the laboratory evolution of *in vitro* artemisinin tolerance by Rocamora *et al*. (2018). Two independent lineages of the 3D7 laboratory line with modestly enhanced ring-stage survival and a measurable trophozoite survival enhancement were generated by cycling of artemisinin drug pressure over 2 years. *Pfk13* mutations were not present, but mutations in *pfap2*α did accrue, as did copy number-dependent upregulation of expression of genes involved in adaptive responses against cellular damage within the parasite, including 6-phosphogluconate dehydrogenase, thioredoxin 1 and a signal peptidase linked to ER-associated degradation. These authors conclude that, in at least one of their parasite lines, resilience to artemisinin derives from an increased capacity to mediate oxidative stress and protein damage, and suggest that a possible mechanism for artemisinin resistance is an enhanced ability of *P. falciparum* to cope with the cellular damage presumably caused by artemisinin directly, and that this could generate K13-independent artemisinin tolerance/resistance in the field. The existence of such parasites is supported by the findings of Mukherjee *et al*. (2017) who identified four *P. falciparum* isolates from Cambodia lacking variant K13, displaying normal clearance dynamics *in vivo* but with enhanced *in vitro* survival in the RSA. Conversely, no direct causative relationship has yet been shown between K13 mutations and enhanced repair of cellular damage.

### Cellular healing—a requirement for parasite survival following artemisinin treatment

**Figure 3.**
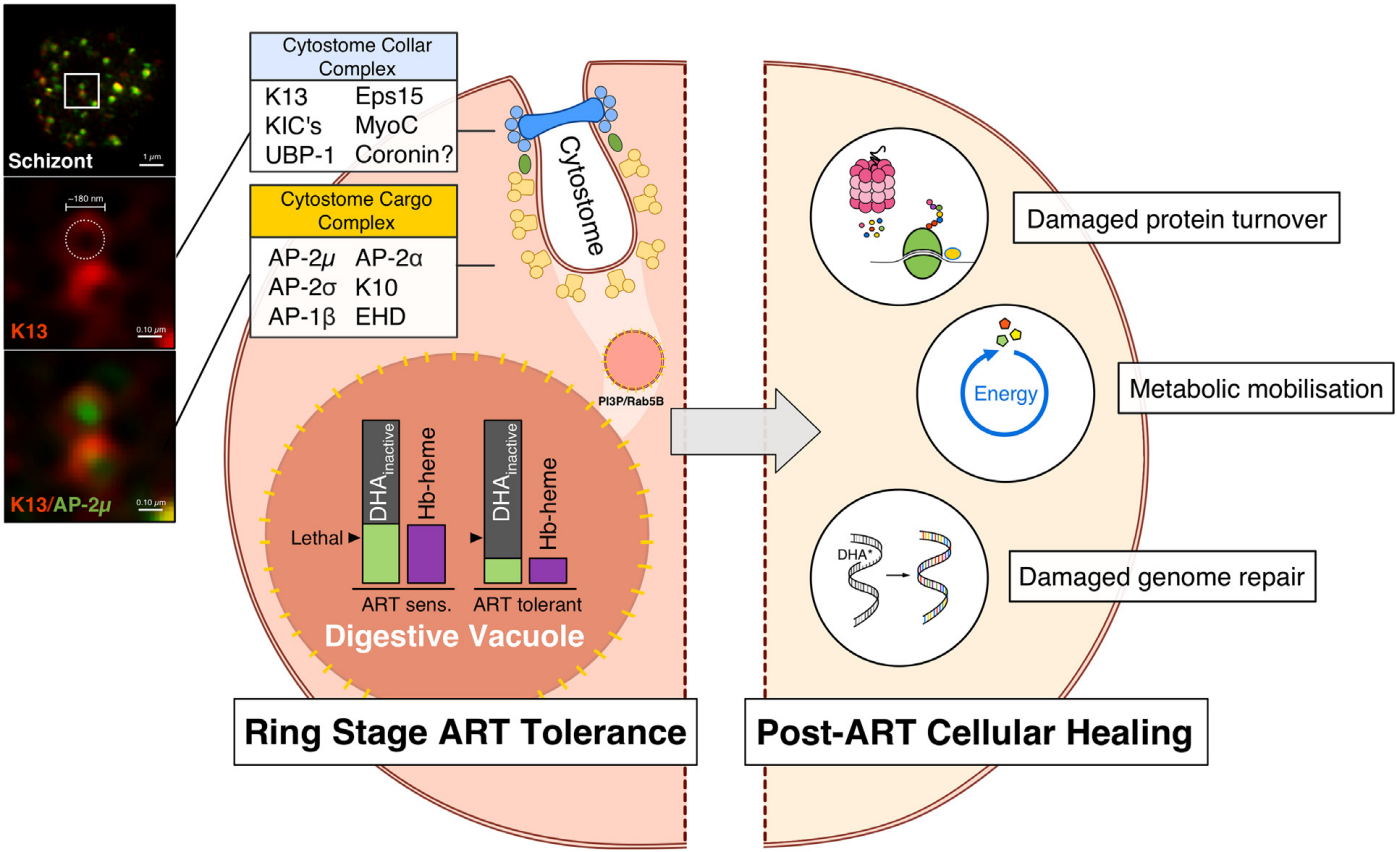
Two-component model of *in vivo* artemisinin tolerance and recovery. *Plasmodium falciparum* survival of artemisinin exposure *in vivo* requires two distinct adaptive components, as even surviving cells are likely to have sustained oxidative damage that would increase the chances of clearance by host defences (O'Flaherty *et al*. 2019). **Left hemisphere:** Recent *in vitro* studies of parasite lineages displaying ring-stage artemisinin-tolerant phenotypes have revealed a common mechanism involving dysregulation of haemoglobin endocytosis via the cytostome. Two complexes seem to be involved: K13-labelled cytostome collar complex (blue) and AP-2-labelled cytostome cargo complex (yellow), which may be linked by KIC7 (green ellipse), a common interacting protein. Two-colour super-resolution SIM imaging (left-most panel; Henrici 2018) of mature schizonts by the authors demonstrates the spatial relationship between these two complexes and recapitulates the ring-shaped K13-defined structure of the cytostome collar with AP-2µ inside this ring in several optical planes. Manipulation of this common pathway leads to a decrease in haemoglobin-derived haem in the DV, and thus a corresponding diminution of activated DHA from supra-lethal concentrations to sub-lethal concentrations (purple and light green bars). This decrease in ring-stage haemoglobin uptake and artemisinin activation allows a fraction of cells to survive. **Right hemisphere:** Surviving cells may have adaptations that mitigate oxidative damage via a variety of as-yet poorly defined pathways. Such enhanced cellular healing phenotypes synergise with the ring-stage artemisinin tolerance phenotype to increase chances of survival in the treated immunocompetent host and enable emergence of artemisinin tolerance in circulating parasite lineages.

The studies summarised above, considered together, suggest that successful artemisinin tolerance *in vivo* leading to enhanced parasite survival in treated patients is the biological sum of two adaptive processes. The first is reduction of ring-stage haemoglobin endocytosis through mutations in loci encoding components of the endocytotic machinery of the ring-stage parasite such as *pfk13*,*pfap2μ*,*pfcoronin* and *pfubp1*, among others (Xie, Ralph and Tilley 2020). The second adaptive component is enhanced response to oxidative stress and protein damage wrought by activated artemisinin in surviving cells (Mok *et al*. 2015; Rocamora *et al*. 2018). In this two-component model (Fig. 2), attenuated endocytosis reduces ring-stage haemoglobin uptake such that early ring-stage parasites encounter a reduced fraction of activated, cytotoxic artemisinin. Functionally impaired endocytosis reduces the concentration of activated artemisinin that the parasite cell sees by transiently starving the cell of haem iron. However, these cells are still affected by the drug molecules that do manage to gain access to Fe^2+^. These activated molecules are terminally reactive warheads that covalently modify proteins. Thus, resumption of cell development and growth requires efficient repair of this damage as well as compensation for fitness costs associated with impaired haemoglobin uptake (Straimer *et al*. 2015). This is particularly important *in vivo* where splenic clearance and innate and acquired host immunity pose additional threats to cell survival in the treated patient (O'Flaherty *et al*. 2019), in contrast to *in vitro* studies, where parasite genetic mutations alone are sufficient to mediate parasite survival in excess of 30%. Further, damaged parasites may not be able to efficiently generate the viable gametocytes, needed for propagation within the circulating *P. falciparum* population. This two-component model for artemisinin resistance (Fig. 3) also provides another plausible explanation for why K13-mediated tolerance has not widely emerged as major public health threat in Africa, although such parasite phenotypes may be present in Rwanda (Uwimana *et al*. 2020). The enhanced damage repair phenotypes prevalent in the GMS, which pre-date the slow clearance phenotype, are necessary for K13 mutants to flourish under drug pressure, but are absent in other endemic regions. It remains unclear how enhanced cellular healing and other ‘background’ phenotypes might interact with non-K13 loci associated with ART tolerance *in vitro* to generate *in vivo* delayed clearance phenotypes in natural populations.

We have a growing understanding of the adaptive mechanisms by which ring-stage Hb endocytosis might be slowed by affecting proteins in either of two inter-linked functional groupings—the first defined by K13 and postulated to reside at the cytostomal neck (Yang *et al*. 2019; Xie, Ralph and Tilley 2020), and the second defined by AP-2 and postulated to reside at sites of endocytosis, which may well be the cytostomal bulb (Fig. 3). Only KIC7 has thus far been implicated as an interactor with both complexes. In contrast, the ‘enhanced cellular healing phenotype’ is barely defined, so how could it be recognised and compared between isolates? Several poorly characterised parasite systems may contribute to enhanced healing, suggested as a prerequisite for clinical emergence and transmission of artemisinin tolerance. Specific mechanisms that deserve attention in future studies are as follows.

- UPR: Are mutations or perturbations in heat-shock proteins, ER-resident chaperones and translational cofactors required for physiologic recovery from ART-induced damage?
- Autophagy: Do autophagy-related genes such as *pfatg18* modulate ART tolerance phenotypes (Breglio *et al*. 2018), and how do autophagosomes and autophagy status contribute to protein turnover and ART-induced oxidative stress response?
- Ubiquitin-proteasome system: Do patterns and mechanisms of ubiquitylation (e.g. K48-polyubiquitylation) differ between sensitive and resistant lineages and contribute to the rate of protein turnover? Are transcriptional changes in proteasomal subunits required for clinical emergence of ART tolerance?
- Metabolism: Does recovery from ART-induced damage require changes in mitochondrial function and energy availability?
- DNA repair mechanisms: To what extent does ART-induced DNA and intranuclear protein or chromatin damage contribute to cytopathology, and how does the cell cope with this damage to resume normal development and faithfully propagate its genome?

Some of these questions can be readily addressed using available tools. *In vitro* methods are available to detect activity levels of proteins implicated in modulating the UPR, such as eIF2α (Zhang *et al*. 2017), and specific inhibitors can also be readily utilised. There also exist well-characterised specific inhibitors of cellular actors along the ubiquitination-proteasomal degradation pathway that could be used as indicators of phenotypic variation in this important system for protein turnover (Turnbull *et al*. 2017). Assays exist to compare DNA repair activity between cell lines, and these have been utilised to measure differences in effectiveness of repair post-artemisinin exposure in *P. falciparum* (Xiong *et al*. 2020). Novel machine learning approaches might also be used to uncover hidden sequence-based features and mutations in these and other factors that correlate with RSA and clinical outcomes to better define the ‘parasite background’ that permits artemisinin tolerance to emerge and spread.

## IMPLICATIONS FOR TREATING *FALCIPARUM* MALARIA *IN VIVO*

We are gaining new insights into the cellular functions of key proteins implicated in modulating the susceptibility of *P. falciparum* to artemisinin *in vitro* and *in vivo*. These proteins are coalescing into two groups as their identities emerge: those that in variant forms, and/or through modified expression levels, elicit impaired ring-stage endocytosis of haemoglobin, so reducing drug activation, and those that contribute to enhanced cellular healing (Fig. 3). Ring-stage parasites with advantageous variants in both groups, thus far described only in the GMS, have an increased chance of surviving artemisinin exposure *in vivo*, although more mature parasites are always killed, and cellular healing of residual artemisinin-induced damage then increases the chances that surviving cells will progress to successful schizogony.

This conceptual model explains two observations concerning *P. falciparum* infections *in vivo* that have puzzled the field. The first is that K13 mutant parasites are completely cleared by extended regimens of artesunate monotherapy and/or ACT (Schallig *et al*. 2017; Sutherland 2017). The second is that K13 propeller domain mutations that induce artemisinin tolerance *in vitro* have not spread widely in African or South American *P. falciparum* populations (Taylor *et al*. 2015; Menard *et al*. 2016; Mathieu *et al*. 2020), possibly because adaptations associated with enhanced cellular healing are rare or absent (Mok *et al*. 2015; Xiong *et al*. 2020). It remains to be seen whether the small focus of K13^R561H^ mutant parasites in Rwanda, which have not yet been shown to display a slow clearance phenotype *in vivo*, represents an important exception (Uwimana *et al*. 2020). There is therefore an opportunity to preserve the effectiveness of ACT for the immediate future by exploiting this growing knowledge of the biological limits of artemisinin tolerance. We recommend the testing of new, imaginative schedules and regimens utilising the combination therapies we already have (van Schalkwyk and Sutherland 2015; Schallig *et al*. 2017). The recent evaluation of the so-called ‘triple ACT’ given over 3 days is a promising step in this direction, although there was no measurable benefit over standard single ACT regimens except in areas where the partner drug piperaquine was failing (van der Pluijm *et al*. 2020). Future studies should compare this approach with combinations given over 6 or 7 days as a means to prevent sub-microscopic and asymptomatic persistence of surviving parasites post-treatment (Beshir *et al*. 2013; Sá *et al*. 2018).

## ACKNOWLEDGEMENTS

We thank Peter Preiser for helpful discussions and access to data prior to publication.
